# Editorial Comment: Techniques - Ultrasound-guided percutaneous nephrolithotomy: How we do it

**DOI:** 10.1590/S1677-5538.IBJU.2020.05.05

**Published:** 2020-07-31

**Authors:** Alexandre Danilovic

**Affiliations:** 1 Hospital das Clínicas da Faculdade de Medicina da USP São Paulo SP Brasil Serviço de Urologia, Hospital das Clínicas da Faculdade de Medicina da USP - HCFMUSP, São Paulo, SP, Brasil

Beiko D ^1^, Razvi H ^2^, Bhojani N ^3^, Bjazevic J ^2^, Bayne DB ^4^, Tzou DT ^5^, et al.

## COMMENT

Ultrasound-guided percutaneous nephrolithotomy (US-PNL) has gained popularity in many parts of the world in the past few years (1). It may be used in combination with fluoroscopy to reduce ionizing radiation exposure or replacing fluoroscopy to completely eliminate exposure to radiation during PNL (2). However, this technique can be challenging to learn.

This article provides some interesting key points for those who would like to start a US-PNL program. Despite other authors claim no different results for obese vs. non-obese patients (3), according to the authors, the ideal candidate to start a learning curve of US-PNL is a healthy non-obese patient whose imaging demonstrates a non-staghorn calculus and at least moderate hydronephrosis. The authors described in details eight steps for a successful US-PNL.

There are many advantages of the use of ultrasound in PNL and this technique should be encouraged. However, some anatomic details of the collecting system are missed by ultrasound. Therefore, it is recommended to progressively convert fluoroscopy to ultrasound guidance step-by-step as one gains experience. More important than completely eliminate exposure to radiation is to keep patient safe from injuries due to suboptimal image guidance.
